# Respiratory Pathogens at Exacerbation in Chronic Bronchitis With Airway Bacterial Colonisation: A Cohort Study

**DOI:** 10.1111/crj.13811

**Published:** 2024-08-20

**Authors:** Thomas L. Jones, Claire Roberts, Scott Elliott, Sharon Glaysher, Ben Green, Janis K. Shute, Anoop J. Chauhan

**Affiliations:** ^1^ Department of Respiratory Medicine Portsmouth Hospitals University NHS Trust Portsmouth UK; ^2^ School of Pharmacy and Biomedical Sciences University of Portsmouth Portsmouth UK; ^3^ Translational Research Laboratory Portsmouth Hospitals University NHS Trust Portsmouth UK

**Keywords:** bacteria, bronchiectasis, COPD, exacerbation, *Haemophilus influenza*, infection, *Pseudomonas aeruginosa*, virus

## Abstract

**Background and Objective:**

COPD and bronchiectasis are common causes of morbidity, particularly around exacerbation. Colonisation with respiratory pathogens can increase the frequency and severity of exacerbations. However, bacterial and viral presence at exacerbation in people with airway colonisation has not been well studied.

**Methods:**

A 6‐month cohort study of participants (*n* = 30) with chronic bronchitis due to bronchiectasis (*n* = 26) and/or COPD (*n* = 13) and colonisation with *Pseudomonas aeruginosa* or *Haemophilus influenzae* was proven on two sputum cultures at exacerbation in the previous 12 months. Participants were provided self‐management education and collected sputum samples daily. Sputum samples at baseline (at least 14 days before or after an exacerbation) and at each exacerbation were examined for a panel of 34 respiratory pathogens using commercially available RT‐PCR kits and compared to results obtained using culture methods for the detection of bacteria.

**Results:**

Participants provided 29 baseline samples and 71 samples at exacerbation. In 17/29 baseline samples, RT‐PCR analysis confirmed the organism demonstrated by culture, while 12 samples showed a discrepancy from culture results. Most exacerbations (57.7%) were not associated with acquiring new bacteria or viruses, while 19.8% showed new bacteria, 15.7% new viruses and 7% both new viruses and bacteria.

**Conclusion:**

Over half of exacerbations were not associated with new organisms in this cohort of participants with chronic bronchitis and colonisation. However, 26.8% demonstrated a new bacterial species in sputum, which is relevant for antibiotic therapy. Baseline RT‐PCR and culture results were discordant in one‐third of participants.

## Graphical abstract

This study investigates airway microbiology at baseline and exacerbation in people with airway colonisation. One‐quarter of exacerbations had a new bacterium present, and one‐quarter had a virus while just over half had no novel organisms at the point of exacerbation.

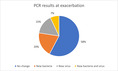

## 1 Introduction

COPD and bronchiectasis [1, 2, 3, 4] are common causes of morbidity worldwide. Exacerbations are a major source of this morbidity [5, 6]; therefore, treating these exacerbations is paramount. People with COPD and bronchiectasis are susceptible to airway colonisation by pathogenic organisms. These organisms include *Pseudomonas aeruginosa* (PA) and *Haemophilus influenzae* (HI), which are common [7] and associated with poor outcomes [8, 9, 10]. Bacterial and viral presence during exacerbations of airway disease has been extensively described [11, 12]; however, this is not the case in the setting of known airway colonisation where organism predominance may affect the dynamics of an exacerbation.

Current optimal treatment of an infectious exacerbation of airway disease in a person with airway colonisation would include antibiotic therapy tailored to the known colonising organism [13, 14]. However, the presence of newly acquired bacteria or viral infections would likely impact the success of this treatment approach, and therefore, the frequency of new bacterial or viral presence is of clinical relevance.

Multiple methods are available for assessment of the airway microbiome. These include culture, which detects viable organisms and is useful for assessment of antibiotic sensitivity but is insensitive, RT‐PCR which is more sensitive, in clinical use and can detect viruses as well as bacterial species, to novel methods such as next generation sequencing which have very high sensitivity but results of uncertain therapeutic and clinical relevance [15].

We aimed to determine the frequency of respiratory pathogens at baseline and exacerbation using sputum RT‐PCR in people with chronic bronchitis due to COPD and/or bronchiectasis, and colonised by HI or PA as assessed by multiple sputum cultures prior to enrolment.

## 2 Methods

We performed a 6‐month prospective cohort study and recruited adults over the age of 18 from our secondary care clinic with chronic bronchitis due to bronchiectasis, COPD or both, between September 2014 and March 2015. Participants were required to have had either PA or HI in sputum culture at exacerbation twice in the previous 12 months, at least one of which must have been in the previous 6 months and not have cultured the other organism in the last 12 months. In addition, participants could give informed consent, comply with study procedures and produce at least 5 mL of sputum most days. A complete study protocol was published detailing recruitment and study procedures [16]. Participants were educated regarding disease self‐management, and a written self‐management plan was provided at the first study visit to ensure that patient self‐management was up to standard of care.

An exacerbation was defined as initiating a course of antibiotics for worsening respiratory symptoms [17, 18], and exacerbation data were captured electronically. Participants managed their exacerbations as per their self‐management plan, with antibiotics provided by their primary care provider or on hospital admission if required. Daily vital signs (pulse rate, SpO2, blood pressure, weight, pedometer step count, peak flow, and temperature) and symptom Visual Analogue Scale (VAS) scores (appetite, breathing, cough, energy, and wellness) were collected electronically daily. Participants were directed to provide sputum samples daily, or as often as possible, in sterile pots and stored in the participants' homes in a −20°C freezer. Samples were collected four weekly and transported in a temperature‐controlled container to central −80°C storage.

Sputum microbiology was assessed with RT‐PCR using samples from enrolment (baseline) and at each subsequent exacerbation ± 1 day. Two commercially available respiratory RT‐PCR kits were used: Respiratory 33 panel (Fast Track Diagnostics Ltd., Luxembourg) and genesig® *Pseudomonas Aeruginosa* RT‐PCR (RegA) (PrimerDesign Ltd., Cambridge, UK). Kits were used according to manufacturers' instructions. A complete list of pathogens detected by the panel is available in the supplement. Detected pathogens were compared to each participant's previous sample, whether the baseline sample or the previous exacerbation, and classified as new or persistent. Dependent on this, exacerbation samples were classed as having new bacteria, new viruses, having both new bacteria and viruses or having neither—no change in detected organisms from the previous sample (baseline or exacerbation).

Software packages used were SPSS 23 (IBM) and Excel 2010 (Microsoft). Results are described as mean and standard deviation for normally distributed data and median (Q1–Q3) for nonnormally distributed data. Comparison of proportions was performed using the Wald test. There was no imputation for missing data. Ethical approval was given by the NHS South Central Research Ethics Committee 14/SC/0298, and all participants gave written, informed consent.

## 3 Results

### 3.1 Baseline Characteristics of the Study Population

Thirty participants were recruited with 29 participants contributing baseline samples and 71 samples at exacerbation. Study recruitment is shown in the CONSORT diagram in Figure S1. One participant died shortly after study enrolment. Participant demographics are shown in Table 1. For participants with bronchiectasis, potential aetiology of the bronchiectasis is listed in Table S1.

**TABLE 1:** Participant baseline data and study exacerbations, values expressed as *n*, mean ± SD, median (Q1–Q3) or percent (%).

		Bronchiectasis (*n* = 17)	COPD (*n* = 4)	Bronchiectasis and COPD (*n* = 9)
Female gender		12, 71%	1, 25%	4, 44%
Age		67.3 (57.8–69.5)	70.2 (61.0–76.4)	73.1 (66.9–75.4)
Exacerbations in year to recruitment		4 (3–4)	4 (2–9)	3 (3–5)
Sputum *P. aeruginosa* (PA) prior to enrolment		9	4	7
Sputum *H. influenzae* (HI) prior to enrolment		8	0	2
Smoking status	Never smoker	10	0	2
	Ex‐smoker	7	4	6
	Current smoker	0	0	1
Pack year smoking history		0 (0–13)	45 (30–70)	32 (18–45)
Spirometry	FEV1%predicted	77.6 ± 28.3	41.3 ± 26.6	54.3 ± 16.2
	FEV1/FVC ratio	77.0 (56.0–80.0)	43.5 (34.5–63.5)	55.0 (49.5–62.5)
BMI		24.8 (21.7–30.5)	23.1 (20.4–27.3)	25.3 (24.6–30.5)
Exacerbations in study (with sputum sample)		2.2 ± 1.5	2.5 ± 1.7	3.3 ± 1.6
Bronchiectasis severity index	Severe	4	—	6
	Moderate	11	—	3
	Mild	2	—	0

### 3.2 Pathogens at Baseline

**FIGURE 1:**
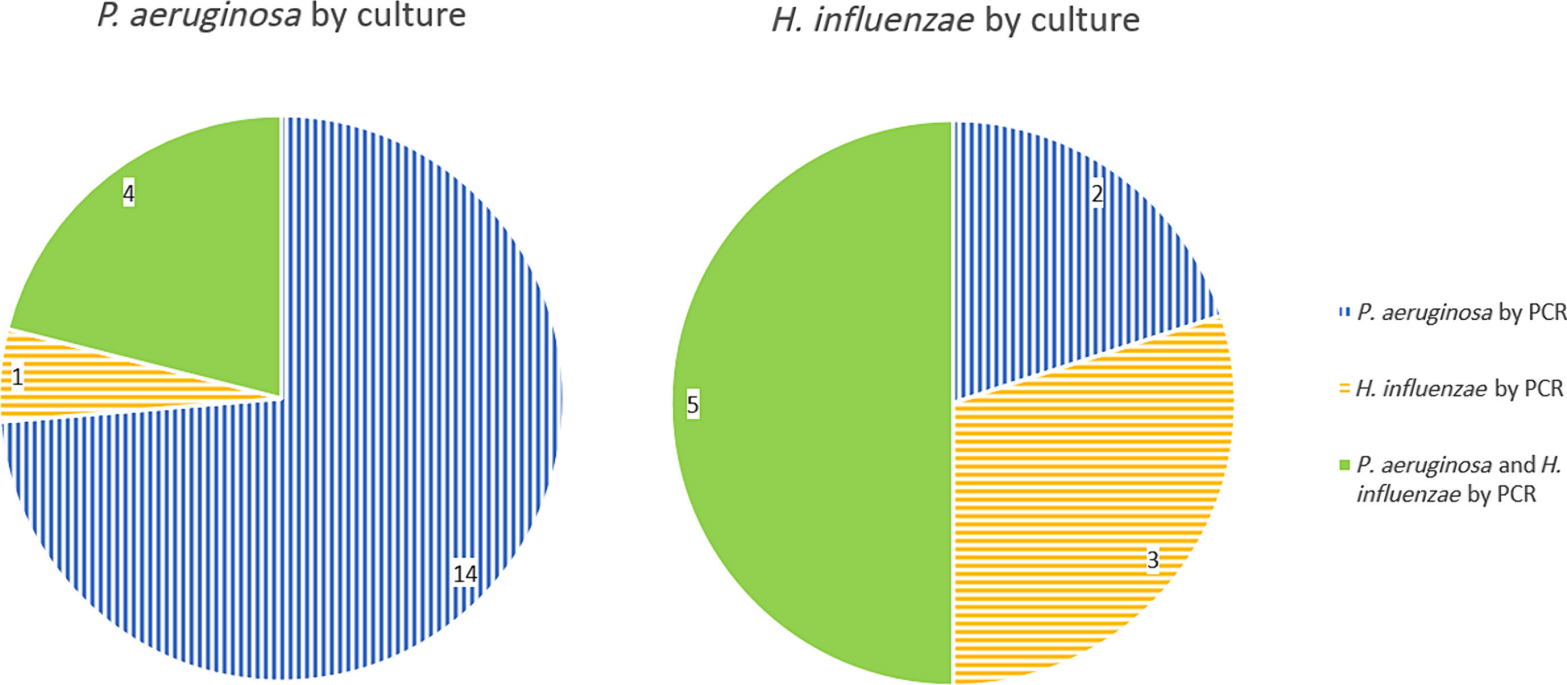
Participant grouping by culture of *Pseudomonas aeruginosa* (*PA*) or *Haemophilus influenzae* (*HI*) before recruitment and by RT‐PCR in baseline samples. PCR, polymerase chain reaction.

**FIGURE 2:**
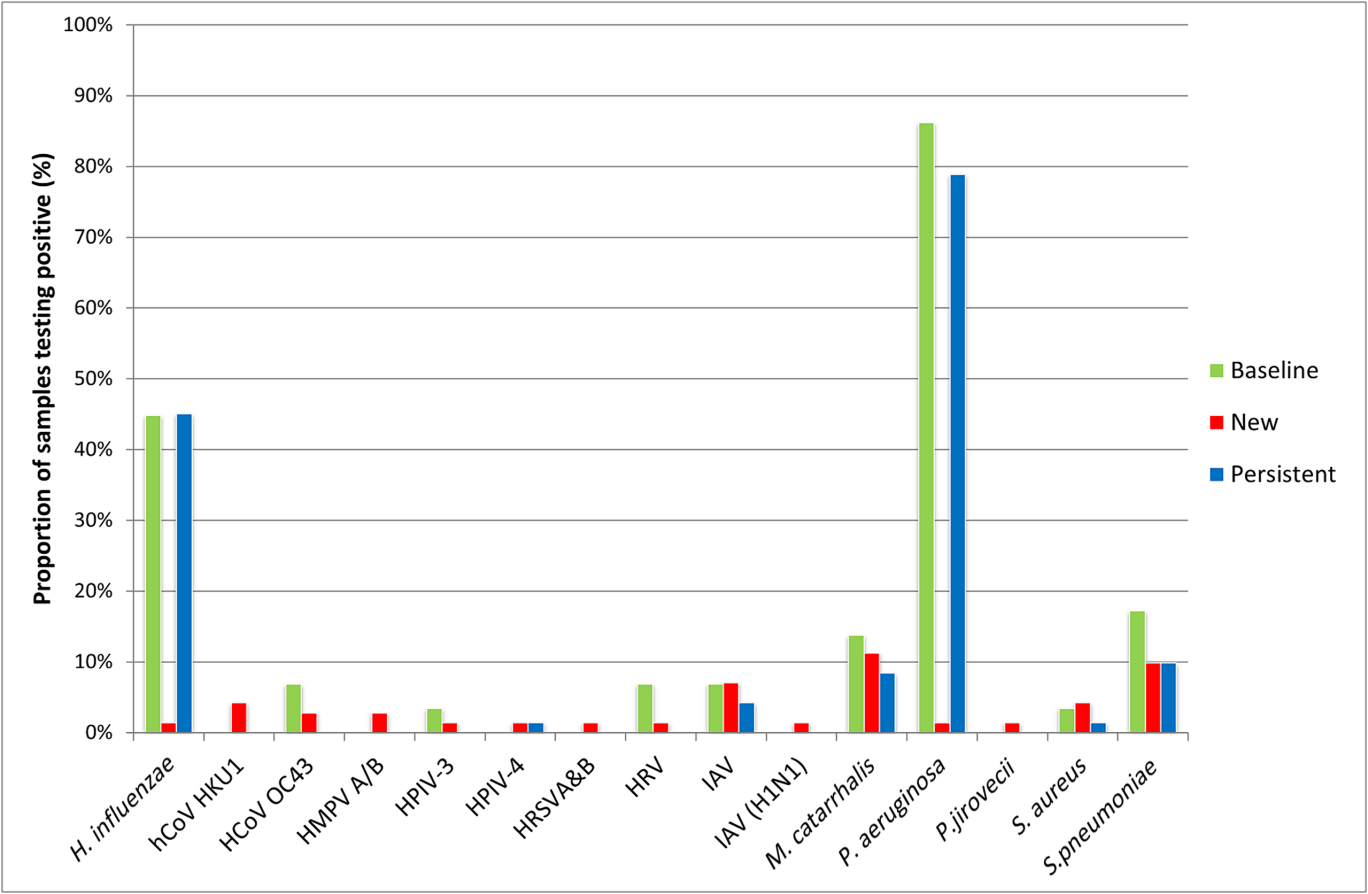
Proportion of baseline and exacerbation sputum samples testing positive for respiratory pathogens by RT‐PCR. Exacerbation samples are labelled as new if the previous sample did not test positive, or persistent if it did. Abbreviations are *Haemophilus influenzae* (*HI*), human coronavirus HKU1 and OC43, human metapneumovirus A/B, human parainfluenza virus 3 or 4, human respiratory syncytial virus A and B, human rhinovirus, influenza A virus and H1N1 strain, *Moraxella catarrhalis* , *Pseudomonas aeruginosa* (*PA*), *Pneumocystis jirovecii*, *Staphylococcus aureus* and *Streptococcus pneumoniae* .

Agreement between RT‐PCR at enrolment and pre‐enrolment sputum culture for presence of *Pseudomonas aeruginosa* (PA) or *Haemophilus* *influenzae* (HI) is shown in Figure 1. Demographics based on PCR grouping are available in Table S2. Repeated sputum cultures in the 6–12 months before enrolment suggested groups of 19 with PA and 10 with HI, but RT‐PCR demonstrated both organisms in 9 participants and the opposite organism in 3 participants with PCR grouping as shown in Figure 1. Baseline samples identified additional bacteria by RT‐PCR; *Staphylococcus aureus* (*n* = 1), *Streptococcus pneumoniae* (*n* = 5) and *Moraxella catarrhalis* (*n* = 4). Viruses detected at baseline were influenza A (*n* = 2), human rhinovirus (*n* = 2), coronavirus OC43 (*n* = 2) and human parainfluenza virus 3 (*n* = 1). Frequency of detection of viruses and bacteria is shown in Figure 2 and Table S4.

### 3.3 Pathogens During Exacerbation

Based on the classification of exacerbations described above, of the 71 exacerbations assessed, 14 (19.8%) were bacterial, 11 (15.5%) viral and 5 (7.0%) had both new bacteria and new viruses. In contrast, 41 samples (57.7%) had no change in detected organisms since the previous sample (baseline or previous exacerbation) as shown in Table S6, suggesting that slightly over half of exacerbations in this cohort were associated with the colonising organism or other causes of exacerbation.

PA was detected in 57 exacerbation samples, of which only one was a new detection and 56 were persistent. *S. aureus* was detected for the first time in three exacerbations and persistently in one. *S. pneumoniae* was detected 14 times at exacerbation of which 7 were new detections. One participant had *Pneumocystis jirovecii* detected in a single exacerbation sample. *M. catarrhalis* was detected in 14 exacerbations of which 8 were new while there was a single new detection of HI and 32 persistent detections. One bacterial exacerbation and one dual bacterial/viral exacerbation demonstrated two new bacterial detections: *S. pneumoniae* and *M. catarrhalis* in one sample and *S. pneumoniae* , HI and influenza A in another sample.

The most commonly detected virus was influenza A, being detected eight times in exacerbation samples of which three were in participants who had previously had it detected (at baseline or the previous exacerbation) raising the possibility of persistent infection. Similarly, parainfluenza 4 was detected twice in subsequent samples in the same participant over 2 months apart. No other viral persistence was noted, with rhinovirus detected at one exacerbation, coronaviruses HKU1 at three and OC43 at two, parainfluenza‐3 being detected once, metapneumovirus twice and RSV once.

The viral detection rate was not significantly different between baseline and exacerbation samples (*p* = 0.84). In addition, there was no significant difference in detection of new bacteria, viruses or both at exacerbation by underlying disease label (*p* = 0.18) or by colonising organism (*p* = 0.37).

Detection of each organism broken down by disease is demonstrated in Table S4, with proportions of viral/bacterial detections per sample shown in Table S5 and classifications of each exacerbation by PCR results in Table S6. Proportions of samples testing positive for viruses or bacteria were compared across disease groups, and no significant difference was found, nor were viruses more likely to be found at exacerbation than baseline.

**FIGURE 3:**
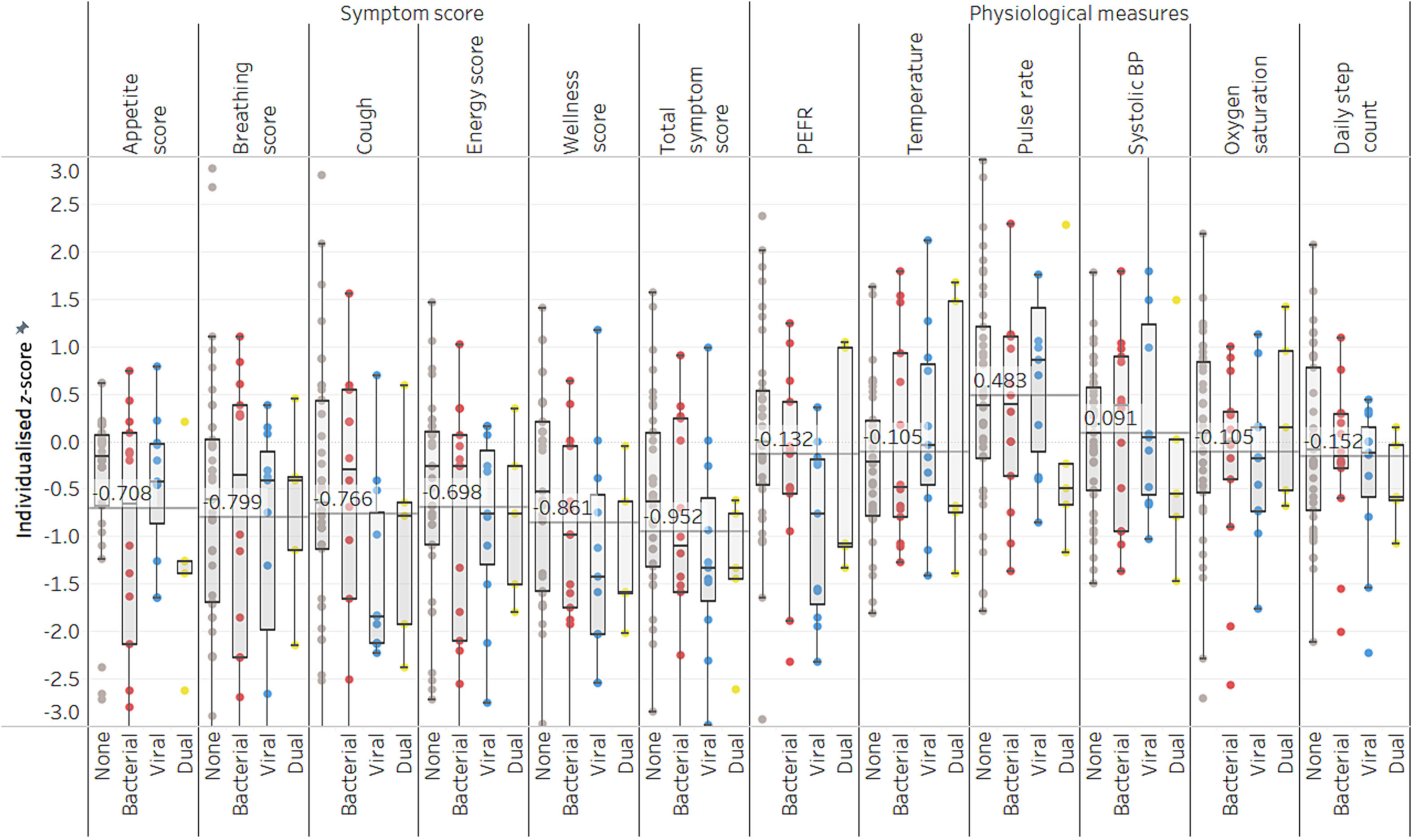
Physiological and symptom data on the day of exacerbation grouped by PCR result categories. Data is shown with median lines for each score/measure, demonstrating a deterioration in symptom scores at the point of exacerbation of less than 1 SD magnitude. No differences between groups based on exacerbation PCR and no significant deterioration of physiological variables are seen.

Physiologic measurements and symptom scores on the day of exacerbation were normalised, and *z*‐scores are shown in Figure 3. These show worsening symptoms at exacerbation without significant changes in physiological variables and no significant differences between groups.

## 4 Discussion

This study demonstrates that, in this population with chronic airway colonisation with PA or HI, over half of exacerbations did not have new organisms detected to account for the worsening symptoms. About 26.8% of exacerbations demonstrated bacteria which were not present in the previous sample suggesting that, in around a quarter of exacerbations, antibiotics targeted at the colonising organism may not be effective. About 15.5% of exacerbations were associated with detection of a respiratory virus as the only change in RT‐PCR detected organisms. Specific viruses such as rhinovirus, parainfluenza‐3 and coronavirus OC43 were more prevalent in baseline samples. In contrast, influenza A, metapneumovirus, coronavirus HKU1 and RSV were more common at exacerbation although there were too few detections to assess the significance of this difference. Overall, the prevalence of viruses was not significantly higher at exacerbation than baseline, and 57.7% of exacerbations did not show new bacteria or viruses. These data suggest that treatment is correct to focus on bacterial infection when people with bacterial colonisation suffer exacerbations; there are no new bacteria present at the point of exacerbation in almost three‐quarters of cases, and guiding management based on previous samples would be appropriate in the majority of exacerbations.

Studies of the microbiome in healthy persons show a diverse range of bacteria, predominantly *Prevotella*, *Veillonella* and *Streptococcus* species which are present in low abundance [19]. In contrast, in COPD and bronchiectasis, bacterial burden is higher but diversity drops in more severe disease with marked microanatomical variation within the same person [12, 20, 21]. People colonised with *Pseudomonas* demonstrate different microbiota than those with *Haemophilus*, but individuals tend to have stable microbiome profiles over time [22]. Previous research and current guidelines state that exacerbations of COPD are largely driven by viruses such as rhinovirus, metapneumovirus, influenza and parainfluenza with smaller contributions from bacteria and other causes of inflammation such as pollution [23, 24]. Microbiome studies at exacerbation in COPD show changes in taxonomic composition, and viral infection may increase bacterial burden [25, 26]. In bronchiectasis, there does not seem to be a consistent change in microbiome at exacerbation, but changes are highly variable between individuals [12, 27].

In this study, we did not find evidence of viral infection frequently at exacerbation in our participants with COPD, rather finding more viruses at baseline than exacerbation, and this may reflect that airway colonisation by pathogenic organisms can cause the inflammation present at exacerbation. Chronic colonisation by organisms such as PA and HI is known to cause inflammation, immune dysregulation and oxidative stress [28, 29]. Acquisition of a new strain of the colonising organism is also associated with exacerbation, but this was not assessed in this study [30, 31]. In bronchiectasis, bacterial infection is known to be part of the pathological cycle driving the disease [32], and the same bacteria are often isolated at exacerbation and baseline [33, 34]. In these data, participants with bronchiectasis were more likely to have a bacterial exacerbation than those with COPD, but almost half of exacerbations were not associated with detection of a new bacterial infection, and viruses were detected as often as new bacteria.

Physiological and symptom data were analysed to determine whether they may be of use in characterising the cause of an exacerbation, but unfortunately, there were no significant differences suggesting they are not of benefit in this area. It is possible that physiological disturbances may become more obvious in untreated cases; however, our participants were educated on self‐management as per standard of care in order to ensure exacerbations are treated early.

The sensitivity of RT‐PCR compared to culture is illustrated by the results demonstrating that, in participants with two cultures of a colonising organism (either PA or HI) in the past 12 months without culture of the other studied organism, 9 out of 29 participants had both organisms detected while 3 participants only had the other organism detected in baseline samples. Clinical management is often guided by culture due to the benefit of antibiotic sensitivity testing, but RT‐PCR has a higher sensitivity for infectious agents [35]. Data is not available to show superiority of management by PCR over culture in exacerbations of airway disease, but speed and sensitivity are both preferable with PCR. The multiplex assay used in this study has previously demonstrated comparable sensitivity to singleplex PCR assays with good specificity [36, 37]. The panel has previously been used to detect coinfection in people with COVID‐19 [38] but does not include a pseudomonas assay. A singleplex pseudomonas RegA assay was therefore used in view of the importance of pseudomonas in this cohort [39, 40].

Newer techniques to detect organisms' nucleic acids are available, loosely termed next generation sequencing (NGS) [41]. These techniques have the ability to map a large amount of the microbiota present, and the respiratory microbiome has been characterised and connected with disease [42]. Unfortunately, the clinical implications and applications of these developments are not yet clear [42]. This study focuses on technologies currently in clinical use, but it is likely that our understanding and treatment of airway infection will evolve as the microbiome becomes better understood and more easily assessed.

This study was a detailed, prospective analysis of exacerbations reflecting real‐world management of patients with chronic bronchitis focussing on those patients with organisms associated with more severe disease. While this 29‐person cohort gives useful information about relative prevalence of infections at the point of exacerbation, there were not enough to make confident assertions about individual organisms. A significantly larger study would be needed for this. As with all nonbronchoscopic studies of airway microbiology, salivary contamination will likely have had an impact on the results. Only organisms within the RT‐PCR panel will have been detected, representing 34 common respiratory pathogens. The mycobiome was not assessed in this study. The study lasted 6 months, and longer studies may provide more useful information, while the study covered the winter period and may therefore be affected by seasonality of infections. This may have increased the likelihood of viral detection in baseline samples. The exacerbations in this study were predominantly diagnosed and self‐managed at home by the participants based on self‐management plans, and the results are likely to be different to a more severe hospitalised population.

## 5 Conclusion

These data are the first to demonstrate the frequency of common respiratory viruses and bacteria in the setting of known bacterial colonisation with PA or HI. Viruses were not detected significantly more often at exacerbation than at baseline with the majority (57.7%) of exacerbation samples tested positive for colonising bacteria only. About 26.8% of exacerbation samples showed new bacteria, and 22.5% had a virus detected. Differences were found between colonising organisms based on culture results and PCR results at baseline. In these data applicable to people colonised with PA or HI, *e*xacerbations were associated with a deterioration of symptoms without significant physiological change, and neither symptom scores nor physiological changes were associated with sputum PCR results suggesting that these cannot be used to predict the presence or absence of bacterial or viral infection.

## Author Contributions

T.L.J. was responsible for methodology, investigation, data curation and writing – original draft preparation. C.R. was responsible for methodology, investigation and writing – review and editing. S.E. was responsible for resources, investigation and writing – review and editing. S.G. was responsible for resources, investigation and writing – review and editing. B.G. was responsible for conceptualization, investigation and writing – review and editing. J.K.S. was responsible for methodology, supervision and writing – review and editing. A.J.C. was responsible for conceptualization, methodology, investigation, supervision and writing – review and editing.

## Ethics Statement

Ethical approval was given by the NHS South Central Research Ethics Committee 14/SC/0298.

## Conflicts of Interest

The authors declare no conflicts of interest.

## Supporting information


**Table S1** Potential causes of Bronchiectasis (multiple causes possible). *Immunodeficiency includes immunodeficient conditions such as immunoglobulin and compliment deficiency and long‐term immunosuppressive medication.
**Table S2.** Participant demographic data at initial study visit, values expressed as mean ± SD, median (Q1–Q3) or percent %. PA, *Pseudomonas aeruginosa* ; HI, *Haemophilus influenzae* ; FEV1, forced expiratory volume in 1 s; FVC, forced vital capacity; BMI, body mass index; COPD, chronic obstructive pulmonary disease.
**Figure S1.** CONSORT diagram of study recruitment. PA, *P. aeruginosa* ; HI, *H. influenzae* .
**Table S3.** Complete list of polymerase chain reaction (PCR) detected organisms.
**Table S4.** Sputum PCR analysis results shown for participants with bronchiectasis, COPD, both and the overall cohort. N = participant count, S = sample count.
**Table S5.** Average number of viral or bacterial detections in baseline or exacerbation samples (e.g., 1.8 indicates a mean of 1.8 detections per sample in that group). Note more organisms detected than samples, mean 1.98 detections of organisms per sample. N = participant count, S = sample count.
**Table S6.** Exacerbation type (by PCR result) for each disease group, divided into no change (same or fewer organisms detected compared to previous sample), new bacteria, new viruses or both.

## Data Availability

The data that support the findings of this study are available from the corresponding author upon reasonable request.
