# Supplementary material for: Respiratory Pathogens at Exacerbation in Chronic Bronchitis With Airway Bacterial Colonisation: A Cohort Study
Source: Clin Respir J. 2024 Aug 20;18(8):e13811. doi: 10.1111/crj.13811 (PMC11333943; doi:10.1111/crj.13811)
Supplement: Supplementary file 1 — Table S1 Potential causes of Bronchiectasis (multiple causes possible). *Immunodeficiency includes immunodeficient conditions such as immunoglobulin and compliment deficiency and long‐term immunosuppressive medication. Table S2. Participant demographic data at initial study visit, values expressed as mean ± SD, median (Q1–Q3) or percent %. PA, Pseudomonas aeruginosa ; HI, Haemophilus influenzae ; FEV1, forced expiratory volume in 1 s; FVC, forced vital capacity; BMI, body mass index; COPD, chronic obstructive pulmonary disease. Figure S1. CONSORT diagram of study recruitment. PA, P. aeruginosa ; HI, H. influenzae . Table S3. Complete list of polymerase chain reaction (PCR) detected organisms. Table S4. Sputum PCR analysis results shown for participants with bronchiectasis, COPD, both and the overall cohort. N = participant count, S = sample count. Table S5. Average number of viral or bacterial detections in baseline or exacerbation samples (e.g., 1.8 indicates a mean of 1.8 detections per sample in that group). Note more organisms detected than samples, mean 1.98 detections of organisms per sample. N = participant count, S = sample count. Table S6. Exacerbation type (by PCR result) for each disease group, divided into no change (same or fewer organisms detected compared to previous sample), new bacteria, new viruses or both. [file CRJ-18-e13811-s001.docx]

|  | **n** | **%** |
| --- | --- | --- |
| **Foreign Body Aspiration** | 1 | 3.8 |
| **Post-pneumonic** | 4 | 15.4% |
| **Rheumatoid arthritis** | 7 | 26.9% |
| **Idiopathic** | 11 | 42.3% |
| **Young’s syndrome** | 1 | 3.8% |
| **ABPA** | 1 | 3.8% |
| **Other airway disease** | 19 | 73.1% |
| **Immunodeficiency*** | 12 | 46.2% |
| **Total** | **26** | **100%** |

Table S1 – Potential causes of Bronchiectasis (multiple causes possible). *Immunodeficiency includes immunodeficient conditions such as immunoglobulin and compliment deficiency and long-term immunosuppressive medication

| Demographics based on PCR grouping | | | | | |
| --- | --- | --- | --- | --- | --- |
|  | | PA (n=16) | HI (n=4) | PA and HI (n=9) | Total (n=29) |
| Female Gender | | 50% | 50% | 77.8% | 58.6% |
| Age (year) | | 66.9 (56.1-71.2) | 67.3 (47.5-73.7) | 71.2(68.4-75.4) | 68.3 (61.3-73.1) |
| Antibiotic courses in previous year | | 4 (3-5) | 4 (3-6) | 3 (2-4) | 4 (3-5) |
| Bronchiectasis | | 87.5% | 100% | 88.9% | 89.7% |
| COPD | | 50% | 25% | 33.3% | 41.4% |
| Smoking status | Never | 35.3% | 75% | 37.5% | 41.4% |
|  | Ex-smoker | 64.7% | 25% | 50% | 55.2% |
|  | Current | 0% | 0% | 12.5% | 3.4% |
| Pack year history | | 19.5 (0-32) | 0 (0-10) | 13 (0-25) | 13 (0-30) |
| FEV1 (%) | | 63.4±26.7 | 74.3±33.5 | 73.9±27.2 | 67.9±27.2 |
| FEV1/FVC ratio | | 55 (43-77) | 71 (61-80) | 73 (56-76) | 65 (52-77) |
| BMI | | 27.3±5.5 | 24.6±5.5 | 25.7±6.0 | 26.5±5.6 |

Table S2 – Participant demographic data at initial study visit, values expressed as mean ± SD, median (Q1-Q3) or percent %. PA – *Pseudomonas aeruginosa*, HI – *Haemophilus* *influenzae*, FEV1 – forced expiratory volume in 1 second, FVC – forced vital capacity, BMI – Body mass index, COPD – chronic obstructive pulmonary disease.


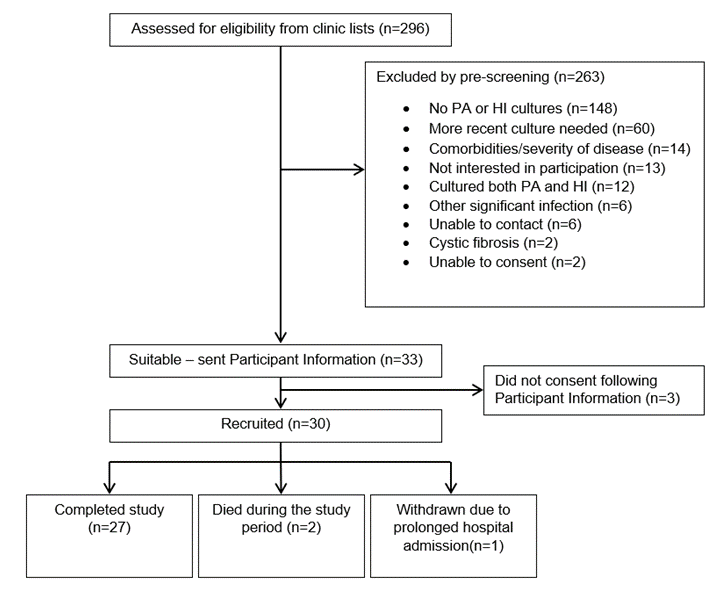


Figure S1: CONSORT diagram of study recruitment. PA – *Pseudomonas aeruginosa*, HI – *Haemophilus* *influenzae*.

| PCR Kit | Pathogens |
| --- | --- |
| Fast Track Diagnostics Respiratory 33 panel | Influenza viruses A, B, C and A (H1N1)  Human parainfluenza viruses 1, 2, 3 and 4  Human coronaviruses NL63, 229E, OC43 and HKU1  Human metapneumoviruses A and B  Human rhinovirus  Human respiratory syncytial viruses A and B  Human adenovirus  Enterovirus  Human parechovirus  Human bocavirus  Pneumocystis jirovecii  Mycoplasma pneumoniae  Chlamydia pneumoniae  Streptococcus pneumoniae  Haemophilus influenzae type B  Staphylococcus aureus  Moraxella catarrhalis  Bordetella spp. except Bordetella parapertussis  Klebsiella pneumoniae  Legionella pneumophila/Legionella longbeachae  Salmonella species  Haemophilus influenzae |
| PrimerDesign | Pseudomonas aeruginosa |

Table S3: Complete list of polymerase chain reaction (PCR) detected organisms

|  |  | Influenza A | Influenza A (H1N1) | Influenza B | Influenza C | Rhinovirus | Coronarvirus 229E | Coronarvirus NL63 | Coronarvirus HKU1 | Coronarvirus OC43 | Parainfluenzavirus-1 | Parainfluenzavirus -2 | Parainfluenzavirus -3 | Parainfluenzavirus -4 | Metapneumovirus A/B | Bocavirus | Respiratory Syncytial virus A&B | Parechovirus | Enteroviruses | Adenovirus | *P. aeruginosa* | *H. influenzae* | *M.pueumoniae* | *S. aureus* | *C. pneumoniae* | *H. influenzae B* | *S.pneumoniae* | *L.pneumophila/ longbeachae* | *K. pneumoniae* | *Salmonella* | *M. catarrhalis* | *Bordetella* | *P.jirovecii* |
| --- | --- | --- | --- | --- | --- | --- | --- | --- | --- | --- | --- | --- | --- | --- | --- | --- | --- | --- | --- | --- | --- | --- | --- | --- | --- | --- | --- | --- | --- | --- | --- | --- | --- |
| **Total**  n=29 | Baseline  S=29 | 2 | 0 | 0 | 0 | 2 | 0 | 0 | 0 | 2 | 0 | 0 | 1 | 0 | 0 | 0 | 0 | 0 | 0 | 0 | 25 | 13 | 0 | 1 | 0 | 0 | 5 | 0 | 0 | 0 | 4 | 0 | 0 |
|  | Exacerbation  S=71 | 8 | 1 | 0 | 0 | 1 | 0 | 0 | 3 | 2 | 0 | 0 | 1 | 2 | 2 | 0 | 1 | 0 | 0 | 0 | 56 | 33 | 0 | 4 | 0 | 0 | 14 | 0 | 0 | 0 | 14 | 0 | 1 |
|  | Total  S=100 | 10 | 1 | 0 | 0 | 3 | 0 | 0 | 3 | 4 | 0 | 0 | 2 | 2 | 2 | 0 | 1 | 0 | 0 | 0 | 81 | 46 | 0 | 5 | 0 | 0 | 19 | 0 | 0 | 0 | 18 | 0 | 1 |
| **Bronchiectasis**  **N=17** | Baseline  S=17 | 2 | 0 | 0 | 0 | 2 | 0 | 0 | 0 | 1 | 0 | 0 | 1 | 0 | 0 | 0 | 0 | 0 | 0 | 0 | 14 | 9 | 0 | 1 | 0 | 0 | 3 | 0 | 0 | 0 | 3 | 0 | 0 |
|  | Exacerbation  S=33 | 8 | 1 | 0 | 0 | 0 | 0 | 0 | 2 | 0 | 0 | 0 | 0 | 0 | 1 | 0 | 1 | 0 | 0 | 0 | 26 | 20 | 0 | 1 | 0 | 0 | 6 | 0 | 0 | 0 | 7 | 0 | 1 |
|  | Total  S=50 | 10 | 1 | 0 | 0 | 2 | 0 | 0 | 2 | 1 | 0 | 0 | 1 | 0 | 1 | 0 | 1 | 0 | 0 | 0 | 40 | 29 | 0 | 2 | 0 | 0 | 9 | 0 | 0 | 0 | 10 | 0 | 1 |
| **COPD**  **N=3** | Baseline  S=3 | 0 | 0 | 0 | 0 | 0 | 0 | 0 | 0 | 1 | 0 | 0 | 0 | 0 | 0 | 0 | 0 | 0 | 0 | 0 | 3 | 1 | 0 | 0 | 0 | 0 | 0 | 0 | 0 | 0 | 0 | 0 | 0 |
|  | Exacerbation  S=10 | 0 | 0 | 0 | 0 | 0 | 0 | 0 | 0 | 1 | 0 | 0 | 0 | 0 | 0 | 0 | 0 | 0 | 0 | 0 | 8 | 3 | 0 | 0 | 0 | 0 | 1 | 0 | 0 | 0 | 2 | 0 | 0 |
|  | Total  S=13 | 0 | 0 | 0 | 0 | 0 | 0 | 0 | 0 | 2 | 0 | 0 | 0 | 0 | 0 | 0 | 0 | 0 | 0 | 0 | 11 | 4 | 0 | 0 | 0 | 0 | 1 | 0 | 0 | 0 | 2 | 0 | 0 |
| **Bronchiectasis**  **and COPD**  **N=9** | Baseline  S=9 | 0 | 0 | 0 | 0 | 0 | 0 | 0 | 0 | 0 | 0 | 0 | 0 | 0 | 0 | 0 | 0 | 0 | 0 | 0 | 8 | 3 | 0 | 0 | 0 | 0 | 2 | 0 | 0 | 0 | 1 | 0 | 0 |
|  | Exacerbation  S=28 | 0 | 0 | 0 | 0 | 1 | 0 | 0 | 1 | 1 | 0 | 0 | 1 | 2 | 1 | 0 | 0 | 0 | 0 | 0 | 22 | 10 | 0 | 3 | 0 | 0 | 7 | 0 | 0 | 0 | 5 | 0 | 0 |
|  | Total  S=37 | 0 | 0 | 0 | 0 | 1 | 0 | 0 | 1 | 1 | 0 | 0 | 1 | 2 | 1 | 0 | 0 | 0 | 0 | 0 | 30 | 13 | 0 | 3 | 0 | 0 | 9 | 0 | 0 | 0 | 6 | 0 | 0 |

Table S4: Sputum PCR analysis results shown for participants with bronchiectasis, COPD, both and the overall cohort. N=participant count, S=sample count

|  |  | **Viruses** | **Bacteria** |
| --- | --- | --- | --- |
| **Total**  n=29 | Baseline  S=29 | 0.241 | 1.655 |
|  | Exacerbation  S=71 | 0.296 | 1.704 |
|  | Total  S=100 | 0.280 | 1.690 |
| **Bronchiectasis**  **N=17** | Baseline  S=17 | 0.353 | 1.765 |
|  | Exacerbation  S=33 | 0.394 | 1.818 |
|  | Total  S=50 | 0.380 | 1.800 |
| **COPD**  **N=3** | Baseline  S=3 | 0.333 | 1.333 |
|  | Exacerbation  S=10 | 0.100 | 1.400 |
|  | Total  S=13 | 0.154 | 1.385 |
| **Bronchiectasis**  **and COPD**  **N=9** | Baseline  S=9 | 0.00 | 1.556 |
|  | Exacerbation  S=28 | 0.250 | 1.679 |
|  | Total  S=37 | 0.189 | 1.649 |

Table S5: Average number of viral or bacterial detections in baseline or exacerbation samples (e.g. 1.8 indicates a mean of 1.8 detections per sample in that group). Note more organisms detected than samples, mean 1.98 detections of organisms per sample. N=participant count, S=sample count

|  | **Bronchiectasis** | **COPD** | **Bronchiectasis and COPD** | **Total** |
| --- | --- | --- | --- | --- |
| **No change** | 16 | 8 | 17 | 41 |
| **New bacteria** | 8 | 1 | 5 | 14 |
| **New virus(es)** | 8 | 1 | 2 | 11 |
| **New bacteria and virus(es)** | 1 | 0 | 4 | 5 |
| **Total** | 33 | 10 | 28 | 71 |

Table S6: Exacerbation type (by PCR result) for each disease group, divided into No change (same or fewer organisms detected compared to previous sample), new bacteria, new viruses or both.
